# In the physical to digital transition with friends – a story of performing inclusive research together no matter what life throws at you

**DOI:** 10.1111/bld.12408

**Published:** 2021-08-02

**Authors:** Lilly Cook, Pedro Rothstein, Lizzie Emeh, Pino Frumiento, Donald Kennedy, David McNicholas, Ifeoma Orjiekwe, Michaela Overton, Mark Snead, Robyn Steward, Jenny M Sutton, Melissa Bradshaw, Evie Jeffreys, Sue Charteris, Sarah Ewans, Mark Williams, Mick Grierson, Dorota Chapko

**Affiliations:** Heart n Soul at The Hub, London, United Kingdom; 1 Creative Computing Institute, University of the Arts, London, United Kingdom

**Keywords:** learning (intellectual) disability, inclusive research, participatory research, research design, digital inclusion

## Abstract

**Background:**

As part of ‘The Hub’ project at Wellcome Collection, a team of eight co-researchers with learning disabilities alongside academics created an online survey to challenge public understanding of learning disabilities. Using creative and arts-based methods, co-researchers remotely co-analysed the survey results amid Covid-19 lockdown challenges. Here, we explore our unexpected ‘transition’ journey from the physical ‘Hub’ to the digital space.

**Methods:**

We organised 20 sessions at ‘The Hub’ and used audio/video/photo recordings to ‘capture’ key moments. With the lockdown, we ensured that every co-researcher had access to and support for digital technologies. Throughout 2020, we organized 28 Zoom meetings involving all co-researchers. In June, Lilly^1^ and Sue^2^ conducted Zoom interviews with the co-research team to reflect on our ‘transition’ journey. In this creative video-form submission accompanied by an accessible report, Lilly puts together a story of how we transitioned and felt throughout this process.

**Findings:**

We identify that trust and the social bonds established at ‘The Hub’ are the key components of our transition to the digital environment. There is the tension between longing for in-person contact and trying to make the most out of the situation to maintain these relationships. At the heart of this is the motivation to ‘change the world’ and the strive for social justice. Having time and opportunity to improve, and co-researchers’ steady growth in confidence, are equally important.

**Conclusions:**

The determination for maintaining friendships among co-researchers and the motivation to ‘change the world’ overcome Covid-19 related challenges in continuing co-research.

**Summary:**

**Table:** 

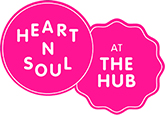	Heart n Soul are an arts organisation who support the creative talents of people with learning disabilities and autistic people.
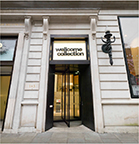	We have been working on a research project called Heart n Soul at The Hub at Wellcome Collection.
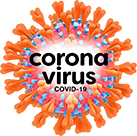	But because of coronavirus, we all had to stay home to stay safe.
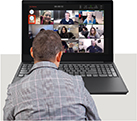	To carry on with our research project we participated in 28 research meetings on Zoom.
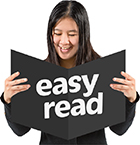	Along with a video, this is an easy read to tell you about how we did it and how we felt about it.

## Background

### Introduction

**Table:** 

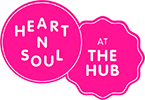	Heart n Soul are an arts organisation who believes in the power and talents of people with learning disabilities and autistic people.
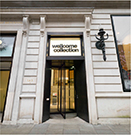	In 2018 we started a research project called **Heart n Soul at The Hub** at Wellcome Collection.
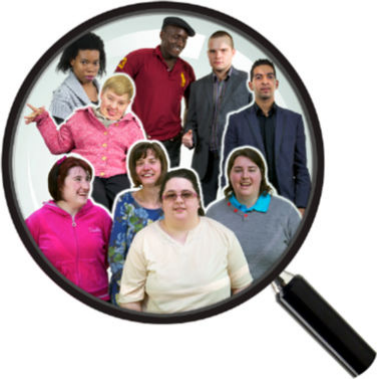	At The Hub we worked with researchers, artists, clinicians, computer scientists and designers to do research.
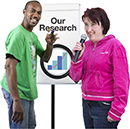	We did a type of research together called ‘inclusive research’.
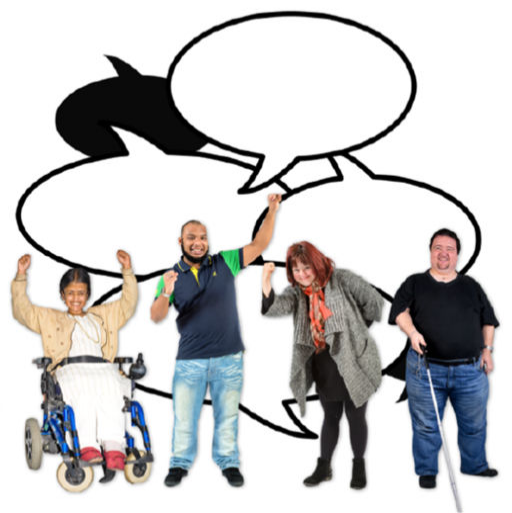	This means research is done in a way that includes people with learning disabilities. We want to lead research and be listened to.

### What we did at The Hub

**Table:** 

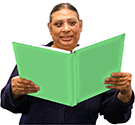	We found that in academic writing attitudes towards people with learning disabilities are generally positive and inclusive.
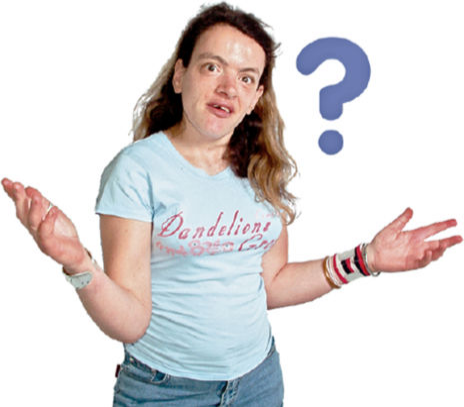	But this did not fit with the experiences of everyone on our project. They did not feel like society has mainly positive feelings towards them.
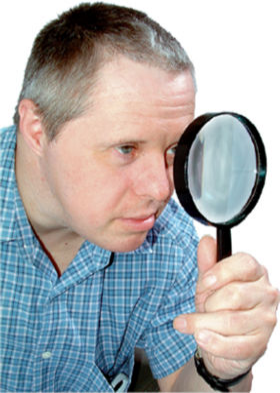	As a team of diverse people we wanted to know why this was happening.
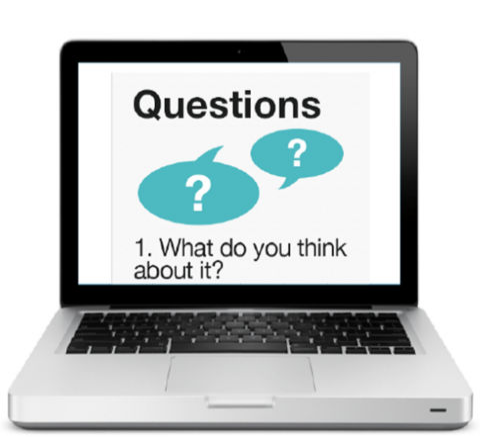	To try and find out more about how people really feel about learning disability and autism we created a new kind of online survey.
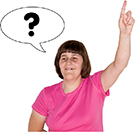	We felt a survey would be a good way to ask questions. But only if we could make it accessible and ask our own questions directly to the public in a creative way.
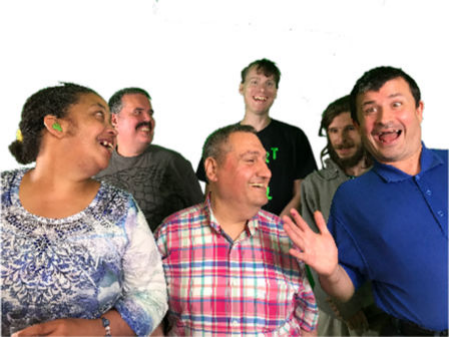	8 co-researchers who enjoyed coming to The Hub the most worked on the survey questions in detail. They were: **Pino, Robyn, Lizzie, Mark S, Ifeoma, David, Michaela, Donald**.
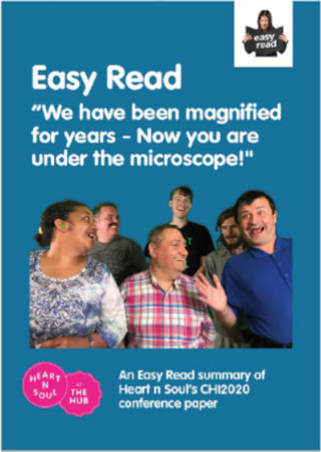	We co-designed our questions and survey together. We wrote a paper about this too! https://dl.acm.org/doi/abs/10.1145/3313831.3376278
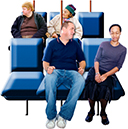	Some questions from our survey included: ◦ People stare at me all the time. What do you see when you see me?
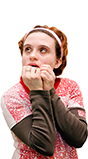	◦ Are you frightened of people with learning disabilities?
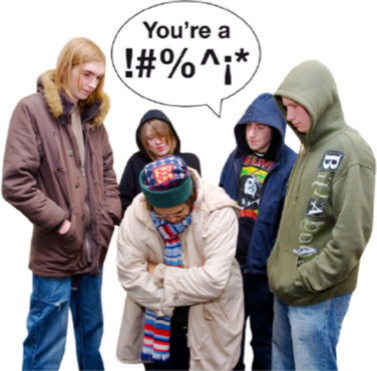	◦ What’s it like to have a learning disability? Some people don’t understand us. Some take the mickey. And I don’t like it. How would you feel if you were in our shoes?
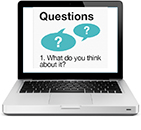	We collected more than 1,500 responses to our first survey!

### Transitioning to working from home

**Table:** 

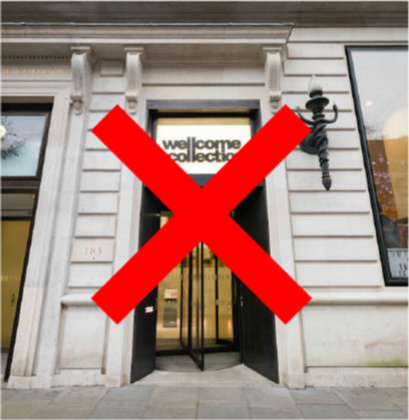	As we were trying to find out what the responses to our survey meant, a lockdown happened. This meant we could no longer visit The Hub.
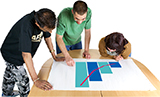	But the lockdown did not stop us from co-analysing our survey responses. Co-analysis means evaluating information together so everyone can contribute.
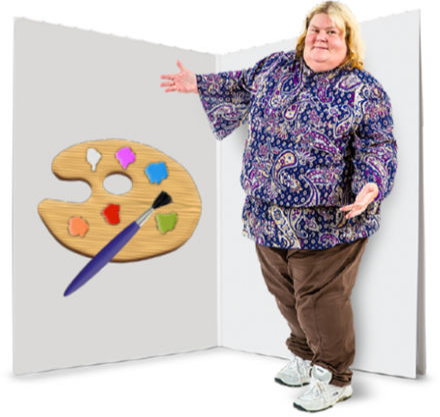	We used creative ways like singing, drawing, painting, plasticine, creative writing to co-analyse the information. All remotely at home!
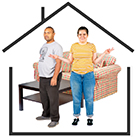	In the next section we will tell you how we did it. We will also tell you how we felt about moving away from The Hub and trying to do co-research remotely from home.

## Methods

### How we did it

**Table:** 

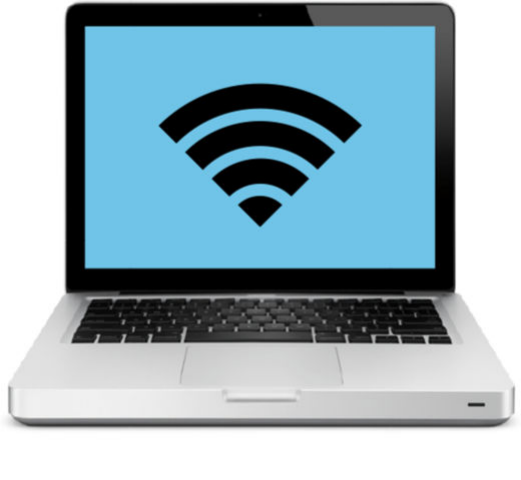	We needed access to computers and the internet. It took some time and team effort to make sure that all of us are comfortable with using Zoom. We tell a story about this is in a different article! https://doi.org/10.1145/3461778.3462010 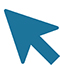
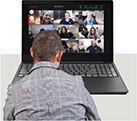	Throughout 2020, we organised 28 Zoom meetings involving all co-researchers.
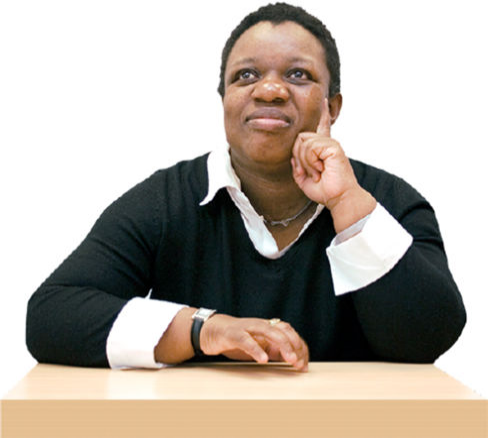	In June, Lilly and Sue conducted a series of Zoom interviews with the team to reflect on our ‘transition’ journey from working at The Hub to online.
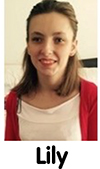	Lilly is a multi-media journalist and a trustee for Heart n Soul. Sue is an independent Leadership Coach and Mentor.
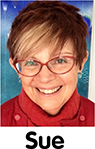	Lilly and Sue asked all members of the research team what they thought of and felt about co-research at that time.
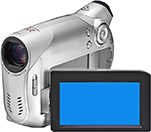	Lilly then made a video using these interviews along with videos filmed when we were working at The Hub. In our film we share some of our experiences of the transition journey as well as the research that we created.
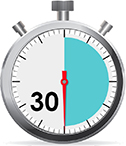	The video is nearly 30 minutes long. You don’t need to watch it all in one go. You can pause it and come back to it.
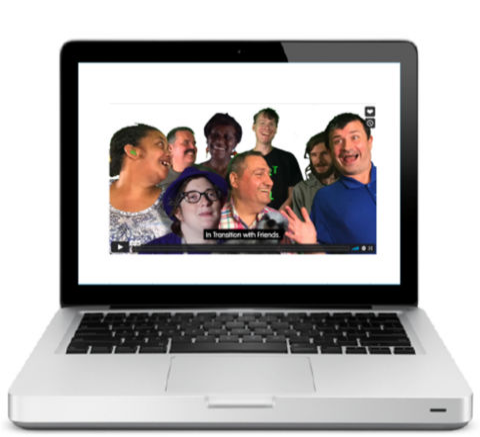	You can watch the video below: 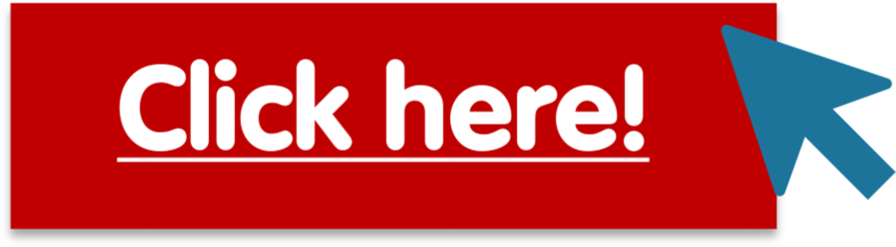 Or type this link into your browser: https://drive.google.com/file/d/1Z67LdJrSyt3Kz9m1_YJaV854QmdiqUOO/view

## Findings

### What we found out

**Table:** 

We came up with 9 themes. We found these important in thinking about our transition journey.	
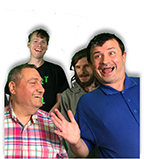	**1. Creative Research** In the video you can see us singing and dancing - these are some of the methods we used for our research at The Hub and when working from home.
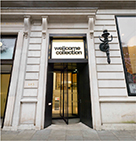	**2.Space in Transition** We miss The Hub! You will hear all of us in the video talk about how important the space was. Happy memories of The Hub kept us motivated and we cherished our friendships.
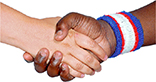	**3. Trust** Our research project could carry on outside of The Hub because we built trust with one another and tried to grow it over the course of the remote sessions.
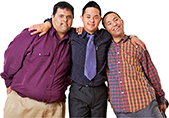	**4.Social Bonds** We are not only co-researchers but we are friends too! During the remote working we wanted to meet to do research together but also to catch-up as friends.
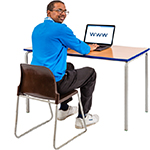	**5. Staying Connected** Staying connected was the key priority for us! None of the team members could stay behind and we made sure that everybody had access to the computer and the internet.
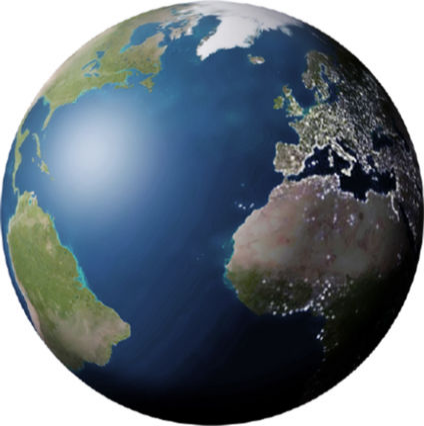	**6. Changing the World** At the heart of this transition is the motivation to ‘change the world’ and the strive for social justice.
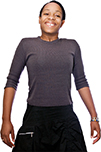	**7.Growing in Confidence** Having the time and opportunity to improve and grow in confidence were important to developing and improving the project.
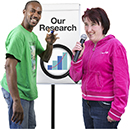	**8.Power Dynamics** Co-researchers took ownership of the project!
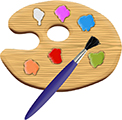	**9. Artwork** Co-researchers created artwork to creatively think about our survey responses and show our research findings in exciting ways!

## Conclusion

### Final thoughts

**Table:** 

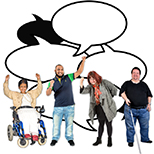	Two things that really kept us going when our research became hard because of the coronavirus was … Our friendships across the team Wanting to ‘change the world’
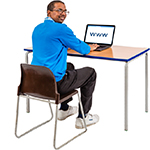	We hope our project has shown that people with learning disabilities can transition well to working online when there is trust and mutual respect.
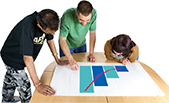	It is also not often that people with learning disabilities have the opportunity to take a detailed look at research results.
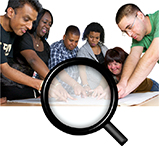	We created new ways of looking at the information from our surveys so that everyone could take part in finding out what the results mean.

## Accessible summary

- We are members of Heart n Soul, an arts organisation who support the creative talents of people with learning disabilities and autistic people.
- We have been working on a research project at ‘The Hub’ at Wellcome Collection in a team consisting of academic and non-academic professionals with diverse abilities.
- Because of coronavirus, we all had to stay home to stay safe. To carry on with our research project we took part in 28 creative research meetings on Zoom.
- In this paper and accompanying video, we will tell you how we did it. We will also tell you how we felt about moving away from ‘The Hub’ and trying to do research remotely from home.
- We hope our project has shown that people with learning disabilities can transition well to working online when there is trust and mutual respect.

## References

[R1] Goodley D, Lawthom R, Liddiard K, Runswick-Cole K (2019). Provocations for Critical Disability Studies. Disability & Society.

[R2] Gratton N (2020). People with learning disabilities and access to mainstream arts and culture: A participatory action research approach. British Journal of Learning Disabilities.

[R3] Kara H (2015). Creative research methods in the social sciences: a practical guide.

[R4] Mannay D (2015). Visual, narrative and creative research methods: Application, reflection and ethics.

[R5] Nind M (2016). Inklusive Forschung Gemeinsam mit Menschen mit Lernschwierigkeiten forschen.

[R6] Ouellette-Kuntz H, Burge P, Brown HK, Arsenault E (2010). Public Attitudes Towards Individuals with Intellectual Disabilities as Measured by the Concept of Social Distance.

[R7] Richards M, Lawthom R, Runswick-Cole K (2019). Community-based arts research for people with learning disabilities: challenging misconceptions about learning disabilities. Disability & Society.

[R8] Strnadova I, Walmsley J (2018). Peer-reviewed articles on inclusive research: Do co-researchers with intellectual disabilities have a voice?. Journal of Applied Research in Intellectual Disabilities.

[R9] Tuffrey-Wijne I, Lam CKK, Marsden D, Conway B, Harris C, Jeffrey D, Stapelberg D (2020). Developing a training course to teach research skills to people with learning disabilities: “It gives us a voice. We CAN be researchers!”.

[R10] Walmsley J (2001). Normalisation, emancipatory research and inclusive research in learning disability. Disability & Society.

